# Hydroclimatic conditions trigger record harmful algal bloom in western Patagonia (summer 2016)

**DOI:** 10.1038/s41598-018-19461-4

**Published:** 2018-01-22

**Authors:** Jorge León-Muñoz, Mauricio A. Urbina, René Garreaud, José Luis Iriarte

**Affiliations:** 1 Interdisciplinary Center for Aquaculture Research (INCAR), Concepción, Chile; 2 Departamento de Zoología, Facultad de Ciencias Naturales y Oceanográficas, Universidad de Concepción, Concepción, 4070386 Chile; 3 Departamento de Geofísica, Universidad de Chile, Santiago, 8370449 Región Metropolitana, Chile; 4 Center for Climate and Resilience Research, CR2, Santiago, 8370449 Región Metropolitana, Chile; 5 Instituto de Acuicultura and Centro de Investigación Dinámica de Ecosistemas Marinos de Altas Latitudes – IDEAL, Universidad Austral de Chile, Los Pinos s/n, Puerto Montt, Chile; 6 Centro COPAS-Sur Austral, Universidad de Concepción, Concepción, Chile; 7 Centro de Investigación en Ecosistemas de la Patagonia (CIEP), Francisco Bilbao 449, Coyhaique, Chile

## Abstract

A harmful algal bloom (HAB) of the raphidophyta alga *Pseudochattonella* cf. *verruculosa* during the 2016 austral summer (February-March) killed nearly 12% of the Chilean salmon production, causing the worst mass mortality of fish and shellfish ever recorded in the coastal waters of western Patagonia. The HAB coincided with a strong El Niño event and the positive phase of the Southern Annular Mode that altered the atmospheric circulation in southern South America and the adjacent Pacific Ocean. This led to very dry conditions and higher than normal solar radiation reaching the surface. Using time series of atmospheric, hydrologic and oceanographic data we show here that an increase in surface water temperature and reduced freshwater input resulted in a weakening of the vertical stratification in the fjords and sounds of this region. This allowed the advection of more saline and nutrient-rich waters, ultimately resulting in an active harmful algal bloom in coastal southern Chile.

## Introduction

The human population is expected to reach ∼8.5 billion in 2030 and to further increase to 9.7 billion in 2050^1^. Feeding that population has been identified as one of the main challenges for our society. One of the most promising alternatives to provide protein for human consumption is aquaculture, although some technical challenges have been identified^2,3^. At the same time that the human population is increasing, climate is changing the long-term statistics and frequency of extreme events^4^, which can affect food security. Climate change also includes direct threats to aquaculture production, as aquatic ecosystems will face an increase in temperature, a decrease in pH and oxygen, salinity fluctuations, and at a broader scale, changes in the circulation patterns and a higher frequency of extreme events^5^. Indeed, some threats such as HABs could be exacerbated^6,7^.

Recent harmful algal blooms along the Pacific coast of North and South America^8^ as well as major lakes have exhibited an unprecedented extent and intensity^9,10^, suggesting that climate change and other drivers are already increasing the risk of these events^11^. These so-called “super blooms” are of great concern due to their large impacts on human health, aquatic ecosystems and economic activities such as aquaculture.

The influence of climate change and variability on freshwater and coastal ecosystems is complex^9,12^, multi-factorial and geographically dependent^13^. In some areas, an increase in rainfall intensity^14,15^ would result in increased nutrient loading to aquatic systems from watersheds subject to land use change^16,17^ and may lead to HABs^12^. In contrast, droughts reduce the freshwater input and hence stratification, which may also alter the hydrobiology of coastal zones^18,19^. Thus there is an urgent need to understand the link between climate, water quality and biological responses on a regional basis^12^.

Chile is the second largest salmon and trout producer worldwide, with an annual export value exceeding US $4.3 billion in 2014. One of the reasons for this success are the diverse types of water bodies (rivers, lakes, estuaries, bays, and fjords) from which the aquaculture industry can benefit in southern Chile^20^. However, this progress has come with an environmental cost, with some severe sanitary and environmental events in recent years^20,21^. This in turn has resulted in significant economic losses for the industry and local communities, as in the 2016 austral summer (January, February, March) when the worst ever mass mortality of fish and shellfish took place in the inner waters of western Patagonia^22^.

**Figure 1:**
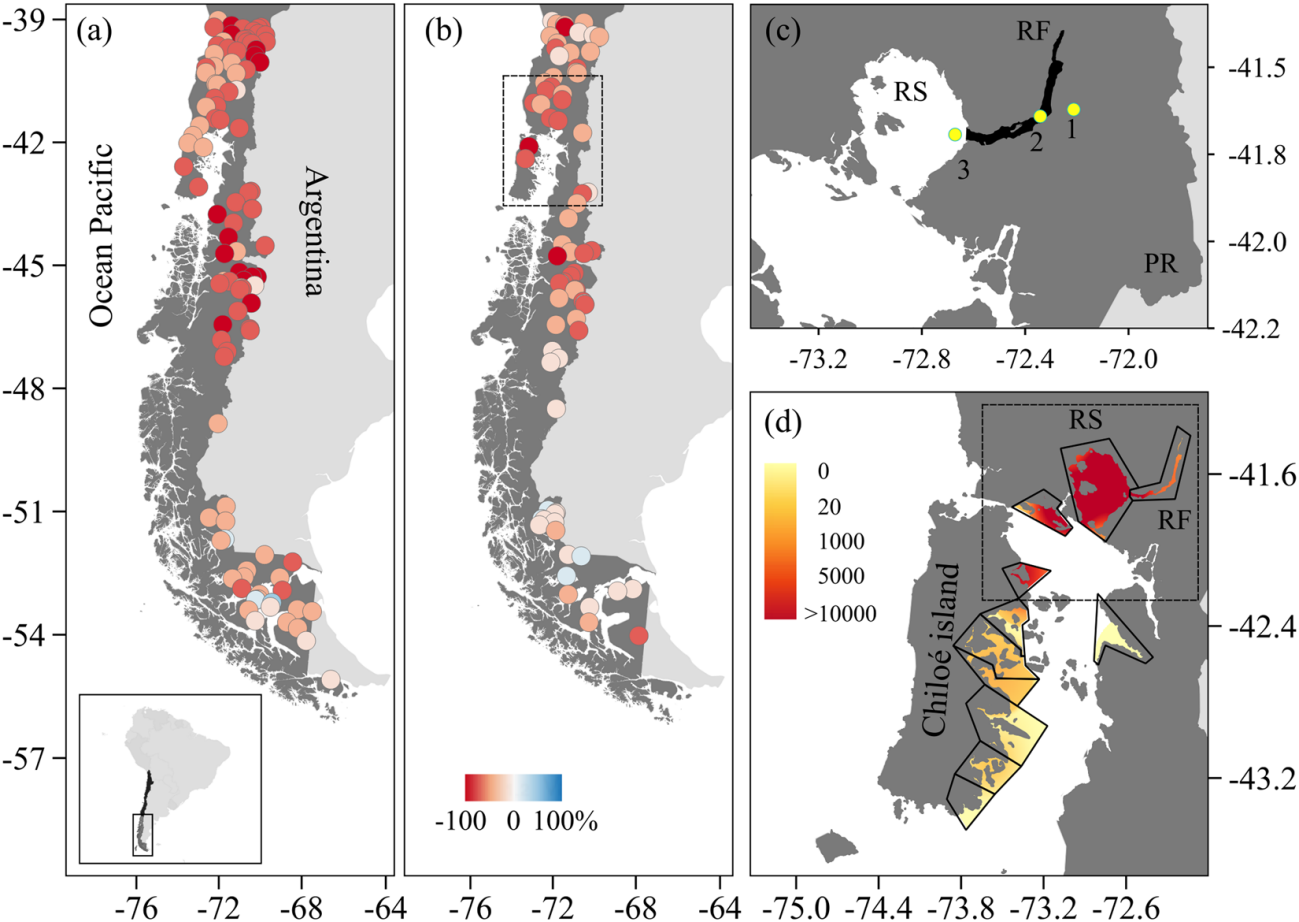
Study region. (**a**,**b**) Chilean Patagonia, coloured circles indicate the accumulated rainfall (**a**) and streamflow (**b**) anomaly (percentage relative to climatology 1980–2010, scale at bottom) during January-February-March 2016. (**c**) Study area: Puelo River (PR) watershed (black line), Reloncaví Fjord (RF) and Reloncaví Sound (RS), where 1 is the PR gauging station, 2 is the RF buoy and 3 is the RS CTD-O station. (**d**) Concentration of *Pseudochattonella* cf. *verruculosa* measured during March 2016 in different salmon farming areas (polygons). Maps were generated using QGIS 2.8.1 https://qgis.org/.

During the austral summer of 2016, a HAB of *Pseudochattonella* cf. *verruculosa* first exterminated nearly 12% of the Chilean salmon production^23^, followed by several fish and shellfish mass mortalities close to Chiloé Island (Fig. 1d), generating a massive economic (>US $800 million losses) and sanitary problem (>40,000 tons of biomass lost)^24^. This was the largest mortality recorded in the salmon industry, only comparable to the losses caused by the Infectious Salmon Anemia virus (ISA) outbreak. To put this in perspective, this HAB produced just in 2 weeks mortality equivalent to that expected in two full years of Chilean salmon production.

*Pseudochattonella* species are cytotoxic raphidophycean flagellates^25^ that cause massive mortality of fishes, especially in fish farms in coastal waters of Scandinavia, New Zealand and Japan^26,27^. The most widely accepted mechanism of these kills is the presence of an icthyotoxin^28^ attributed to free fatty acids^29^, an enhanced production of reactive oxygen species^30^ and other chemical compounds called phycotoxins such as brevetoxins and karlotoxins^31^. The effect of these toxins alone could kill fish within a few hours, but at the same time gill damage (impairing ion exchange) and blockage (impairing gas exchange) could certainly worsen this deadly effect.

Current research efforts have focused on determining the possible causes of this last severe HAB event, but what triggered the last HAB and the reasons for its severity are still unknown. To address this gap, this study tested the hypothesis that the interaction of large scale climate and oceanographic anomalies imposed an external influence on *Pseudochattonella* cf. *verruculosa* bloom dynamics (initiation, maintenance, and decline) in southern Chile. This contribution explores some of the potential mechanisms that could have triggered this last HAB, using time series of atmospheric and oceanographic data and examining the concurrent climate forcing (El Niño and the Southern Annular Mode) and local abnormal hydro-biological conditions. This integrated approach to understand the influence of climatic-oceanographic coupling on microorganism growth rates allows better comprehension and prediction of noxious phytoplankton blooms that is particularly relevant in a changing climate.

## Methods

### Study region

We analysed oceanographic and hydrobiological data of the Reloncaví Fjord and Sound, one of the best studied in western Patagonia (Fig. 1c). Reloncaví Fjord and Sound were pioneering areas in salmon farming and one of the most severely affected by the 2016 HAB (Fig. 1d). The circulation in the Fjord and Sound is largely regulated by freshwater input from the Puelo River, which drains a trans-Andean watershed and empties into the middle of Reloncaví Fjord. With an annual average streamflow of 650 m^3^ s^−1^ and a pluvio-nival regime, the Puelo River reaches its maximum streamflow in winter (rainfall) and spring (snowmelt)^18^.

Streamflow of the Puelo River is significantly correlated with the streamflow of other rivers that drain into the middle (Cochamó River *Q* = 100 m^3^ s^−1^) and head (Petrohué River *Q* = 350 m^3^ s^−1^) of the Reloncaví Fjord and with the other main tributary rivers of the coastal systems in western Patagonia (Yelcho River *Q* = 360 m^3^ s^−1^, Palena River *Q* = 130 m^3^ s^−1^, Cisnes River *Q* = 240 m^3^ s^−1^, Aysén River *Q* = 630 m^3^ s^−1^)^32^.

### Data sources and analysis

Station-based precipitation and streamflow data were obtained from the Climate Explorer (http://explorador.cr2.cl/) that compiles quality-controlled records from the Chilean Weather Service and Water Agency. Of particular interest, daily mean streamflow data were obtained from the hydrological station Carrera Basilio (41.6°S; 72.2°W; Fig. 1c), the gauging station closest to the mouth of the Puelo River in the Reloncaví Fjord (Fig. 1c), from 1950 to date. Monthly precipitation data were obtained from the meteorological station of Puerto Montt (41.4°S, 73.1°W). The large-scale state of the atmosphere was characterized using monthly means of sea level pressure (SLP), downward flux of short wave radiation at the surface and wind at selected pressure levels from the National Centers for Environmental Prediction (NCEP)-National Center for Atmospheric Research (NCAR) Reanalysis^33^, available from 1948 onwards on a 2.5°× 2.5° latitude-longitude grid. Sea surface temperature (SST) was obtained from NOAA high-resolution blended SST^34^ from 1981 onwards, also on a 0.25°× 0.25° latitude-longitude grid.

Abundance and composition of phytoplankton species during the HAB were obtained from the Phytoplankton Monitoring Program database of the Research Salmon Institute (INTESAL). This program collects discrete quantitative samples from the surface photic layer in the coastal areas where salmon farming occurs (http://mapas.intesal.cl/publico).

Sampling covers the whole year with emphasis on spring, summer and fall seasons. Weekly-daily samples for phytoplankton were taken from the surface layer during bloom seasons and analysed for total cell abundance using standard inverted microscopy as described by Iriarte *et al*. (2016)^19^.

Hourly data of dissolved oxygen (ml L^−1^), temperature (°C) and salinity (psu) were obtained from a buoy deployed at 4 m depth located adjacent to the Puelo River (Fig. 1c). This “North Patagonia” buoy was deployed on 1 January 2015 at 41°38´S, 72°20´W, equipped with SAMI *p*CO_2_, SAMI-pH (Submersible Autonomous Moored Instrument), temperature, salinity and dissolved oxygen (SeaBird) (see details in the Global Ocean Acidification Observing Network: http://portal.goa-on.org/Explorer). CTD-O profiles in Reloncaví Sound were also sourced from the Fisheries Development Institute of Chile (Fig. 1c). To quantify the stratification, CTD data were processed with Ocean Data View (ODV) software to obtain the Brunt–Väisälä frequency (https://odv.awi.de) as an estimate of the water column stability^35^. We also calculated the sea water density following Fofonoff and Millard (1983)^36^.

The trends of the streamflow and precipitation series were analysed using the non-parametric Mann-Kendall trend test and the regression of the Sen slope^37,38^. To evaluate the behaviour of the Puelo River during the summer of 2016 we generated a flow duration curve with the streamflow data gathered between 1950 and 2016. This approach is commonly used to analyse streamflow series with large data sets^19,39^.

## Background

### Climate context

A trademark of Patagonia’s climate is the copious precipitation (> 3000 mm year^−1^) over its western (Pacific) side delivered by mid-latitude storms embedded in the Southern Hemisphere westerly wind belt and enhanced by the forced uplift over the austral Andes^40^. Consistently, precipitation variability over Patagonia at inter-annual and longer scales is largely explained by concomitant changes in the intensity of the westerly flow impinging on South America between 40–50°S^41,42^ and tied to SLP anomalies. Both El Niño Southern Oscillation (ENSO) and the Southern Annular Mode (SAM) can modulate the SLP/wind fields over the southeast Pacific^43^.

When ENSO is in its warm phase (El Niño condition) during the austral summer, SLP increases and the westerlies weaken off the southern tip of the continent, leading to drier than average conditions in western Patagonia^44^. SAM is the leading mode of atmospheric variability in the SH south of 30°S^45^, characterized by a latitudinal vacillation of the tropospheric-deep westerly wind maxima around 50°S. During its positive phase, the westerlies intensify around the Antarctic periphery and weaken around 40°S, thus reducing precipitation and increasing air temperature over western Patagonia^43,46^. A robust trend of SAM towards its positive polarity during spring and summer has been observed for the last 3–4 decades^47^ and is attributed to the effects of stratospheric ozone depletion and increased greenhouse gas concentrations^48^. The SAM trend is consistent with a contemporaneous decrease in precipitation and streamflow, mainly in summer and autumn, revealed by direct observations (e.g. Fig. 3) and tree-ring based reconstructions in western Patagonia^32,49–52^. Thus anthropogenic global climate change seems to be already altering the climate of Patagonia, mediated by SAM-related changes in circulation and precipitation.

### Hydrobiology

Coastal systems in western Patagonia are heavily influenced by freshwater inputs, directly by rainfall and indirectly through tributary rivers^53^ which are fed by copious precipitation over the austral Andes (Fig. 3). In this unique and highly productive system, the elevated freshwater inputs cause an almost permanent stratification of the water column due to the strong vertical and horizontal salinity gradients^54–56^. In turn, the vertical stratification limits nutrient exchange between the surface layer (mainly fresh and brackish water) and deep layers (mainly of oceanic origin). Although there are no inorganic nutrient data available for 2016 in the area, data from summer 2015 at 15 m depth below the halocline showed nutrient-rich deep waters (nitrate: 10–22 μM; orthophosphate: 0.8–1.8 μM; silicic acid: 30–50 μM, J.L. Iriarte, unpublished). González *et al*. (2013)^57^ found high concentrations of dissolved silicic acid (from tributaries) in the near surface layer, and high concentrations of orthophosphate and nitrate (from sub-Antarctic waters) below the upper freshwater layer. Fjords in this region are characterized by seasonal fluctuations in primary production modulated by the interplay of surface freshwater contributions and deep oceanic water sources^58^.

Inorganic nutrients and radiation have been generally recognized as limiting factors in cold waters, where nitrogen (e.g. nitrate) has been observed to be the key nutrient for high primary productivity in northern Patagonia (0.5–3 g C m^−2^ d^−1^)^59^, mainly dominated by diatom species (90%)^57,58,60,61^.

## Results

### The record-breaking 2016 summer

**Figure 2:**
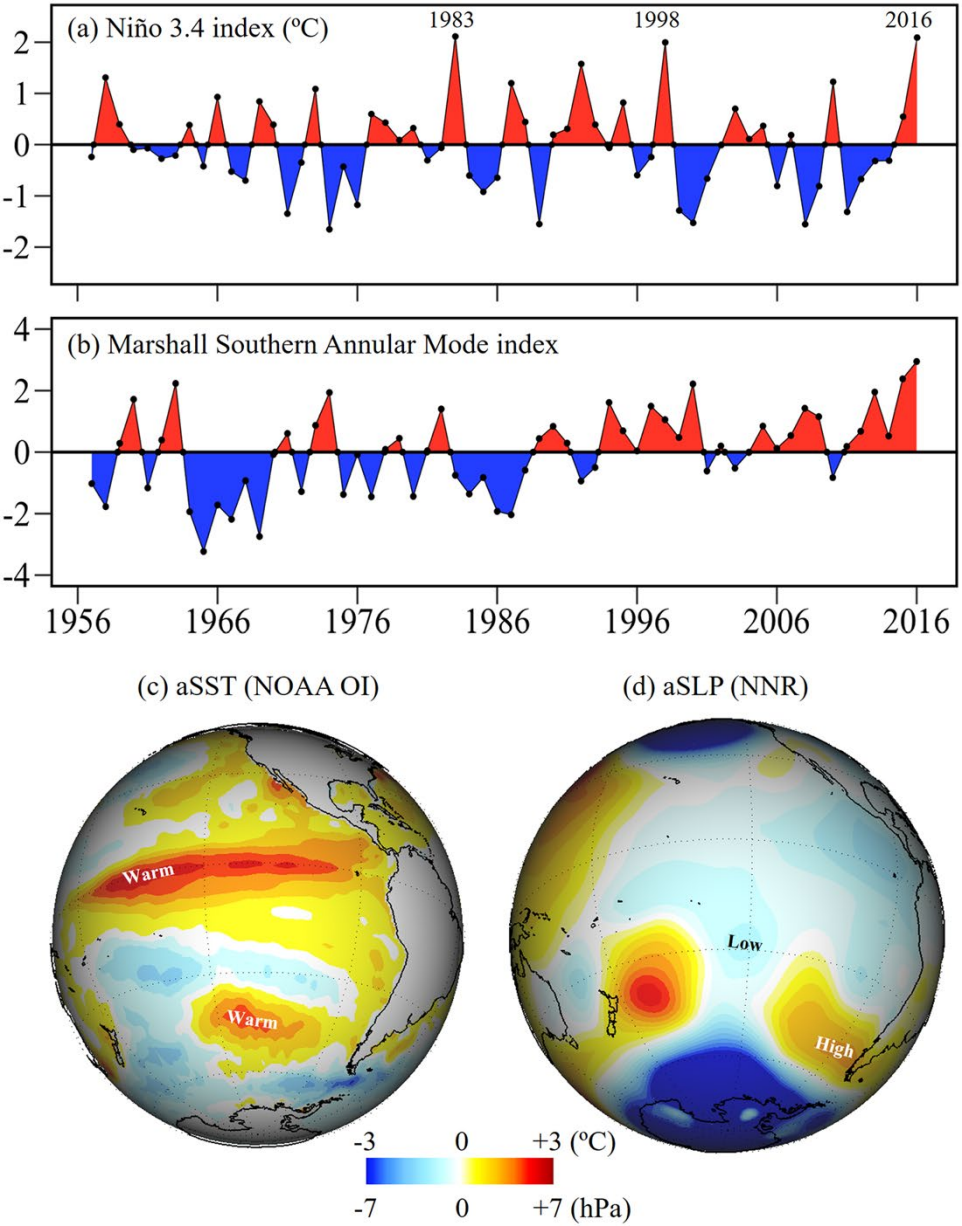
Large scale context during the austral 2016 summer (JFM). (**a**) Niño 3.4 index. (**b**) Marshall Southern Annular Mode (SAM) index. (**c**) Sea surface temperature (aSST) anomalies. (**d**) Sea level pressure (SLP) anomalies. Anomalies are calculated as the seasonal departure from the long term mean. Figure a,b were generated using R 3.3.0 software https://r-project.org/. Figure c,d were generated using the Integrated Data Viewer (IDV5.2) software http://unidata.ucar.edu/software/idv/.

Since 2011 cold conditions had prevailed in the tropical Pacific until a rapid warming began in late 2014, leading to a strong El Niño event by mid-2015^62^. The Niño 3.4 index reached +2.1 °C during austral summer 2016 (JFM), the second highest value since 1948, just below the value in the summer of 1983 and above 1988 (Fig. 2a). Indeed, the large-scale conditions at low latitudes in summer 2016 were typical of an El Niño event, as shown by the anomaly maps of SST and SLP (Fig. 2c,d). The warming across the tropical Pacific exceeded +1.5 °C and affected the southeast Pacific down to Patagonia, where SST anomalies were about +0.5 °C. The SLP field features the typical decrease over the SE Pacific at lower latitudes and a ridge of higher pressure over the south Pacific (50°S). The latter is caused by a Rossby wave train emanating from the tropics^63^ and causes the weakening of the westerly winds over southern South America.

**Figure 3:**
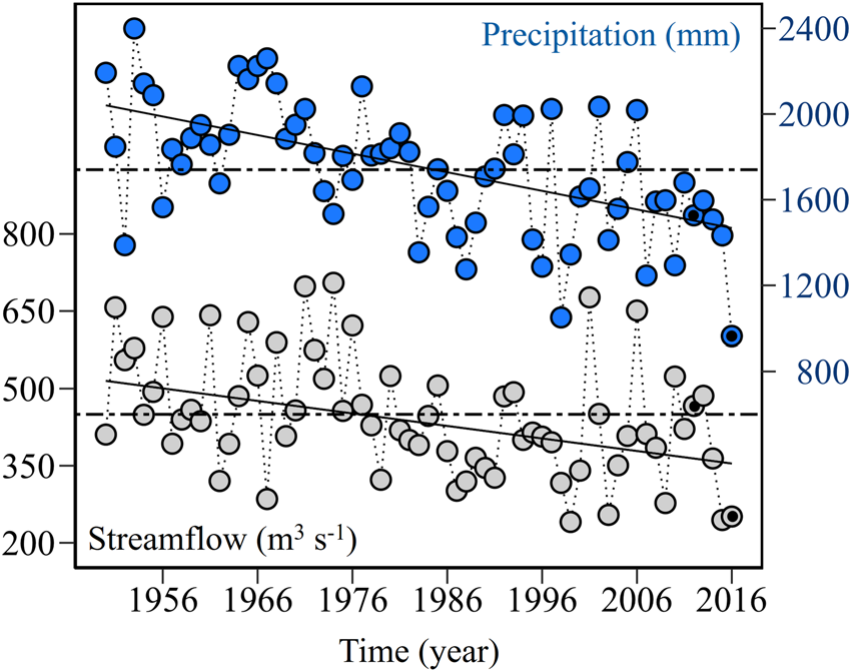
Freshwater input. Significant (p < 0.001) decline of the Puelo River (41.6°S, 72.2°W) summer streamflow (January, February, and March, Sen slope estimate = −2.436 m^3^ s^−1^year^−1^) and Puerto Montt annual precipitation (41.4°S, 73.1°W, Sen slope estimate = −8.682 mm year^−1^) between 1950 and 2016. The values for 2012 and 2016 are highlighted in dark to compare between a “normal” (2012) and a “dry” year (2016) (see also Fig. 6). The trend of the streamflow and precipitation was analysed using the non-parametric Mann-Kendall trend test and the regression of the Sen slope. The dotted line shows the historic average between 1950 and 2016. Figure generated using R 3.3.0 software https://r-project.org/16.46 20.22.

While the strong El Niño was undoubtedly instrumental in the maintenance of the anticyclonic ridge over the south Pacific/South America, SAM also played a role as it reached its highest value during the summer of 2016 (Fig. 2b), associated with mostly positive SLP anomalies in mid latitudes and very negative anomalies at higher latitudes (Fig. 2d). The fact that both modes were in their positive state during the summer of 2016 is surprising, considering that El Niño conditions favour the negative phase of SAM, thus producing a negative correlation between their indices^64,65^ at interannual time-scales. Anthropogenic climate change^48,66,67^, however, has been reported to cause a tendency in SAM toward its positive polarity. The elevated values of the SAM index in 2016 could be associated with this positive SAM trend, suggesting that climate change may have had enough effect to overcome the opposing El Niño forcing^68^ during the summer of 2016. We thus posit that SAM (whose trend is linked to anthropogenic forcing) provided a significant circulation system (positive SLP anomalies at midlatitudes) upon which the strong ENSO-related anomalies could have been superimposed, producing the marked ridge off austral Chile and hence the extreme dry conditions over Patagonia (see Garreaud 2018 for an in depth climate analysis).

### Event overview

**Figure 4:**
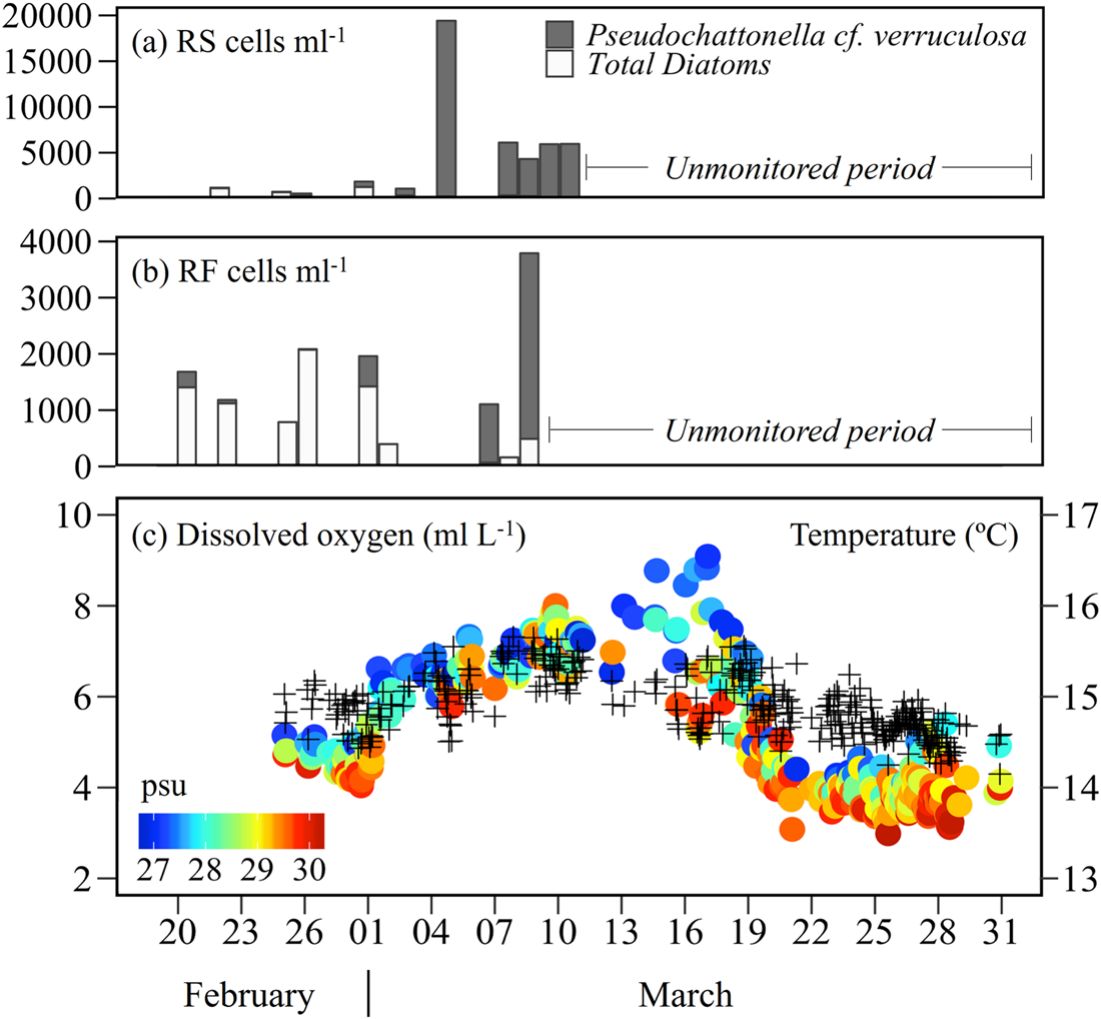
HAB 2016. (**a**,**b**) and *Pseudochattonella* cf. *verruculosa* concentration (cells mL^−1^) between February and March 2016 in Reloncaví Sound (RS) and Reloncaví Fjord (RF), respectively. (**c**) Time series (February – March 2016) of hourly data of dissolved oxygen (DO ml L^−1^ circles), temperature (°C, crosses) and salinity (psu, observations are noted by coloured gradient along the DO time series). Figures generated using R 3.3.0 software https://r-project.org/.

During the 2016 HAB, *Pseudochattonella* cf. *verruculosa* reached concentrations higher than 3 × 10^3^ cells mL^−1^ (up to almost 20 × 10^3^ cells mL^−1^) and made up 95% of the total phytoplankton assemblage in the Reloncaví Fjord and Sound (Fig. 4a,b). During this period diatom cell numbers declined steadily (<500 cells mL^−1^) and the phytoplankton assemblage became progressively dominated by *Pseudochattonella* cf. *verruculosa* (Fig. 4a,b). The transition between these phytoplankton functional groups (diatoms *versus* raphidophytes) has been already reported in other *Chattonella* spp. blooms^26^. After this period, a large late summer bloom (March - April) of the dinoflagellate species *Alexandrium catenella* was observed in the oceanic area adjacent to our study region, associated with large-scale atmospheric and oceanographic processes as has been suggested by Hernandez *et al*.^69^.

### Local conditions

**Figure 5:**
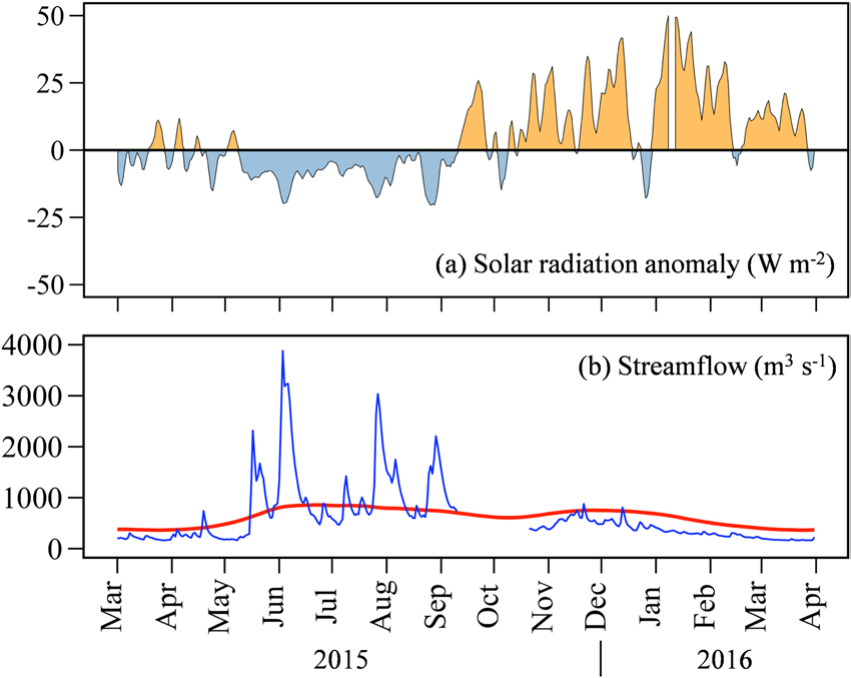
Anomalies of solar radiation and Puelo streamflow 2015–2016. (**a**) Solar radiation anomaly reaching the coastal systems in western Patagonia expressed as a percentage of the observed daily mean value relative to the monthly long-term mean (1980–2010), both in units of Wm^−2^. (**b**) Observed Puelo River streamflow (blue line) and long-term mean streamflow (red line). Figures generated using R 3.3.0 software https://r-project.org/.

Consistent with the large-scale climate forcing mentioned above, drier than normal conditions were observed across southern Chile both in the rainfall and streamflow records (Figs 1a,b and 3). Puelo River streamflow showed a sustained decrease over time, with the values of 2016 below the historical record (Figs 3 and 5b) and even in the context of the last four centuries^51^. For example, in March of 2016 Puelo River streamflow was less than half of the historical average streamflow between 1950 and 2016 (175 m^3^ s^−1^ *vs*. 360 m^3^ s^−1^; Fig. 5b).

At a daily time scale, some of the lowest summer streamflows over the last 66 years were recorded in late March, 2016. The prevalence of higher than normal atmospheric pressure and lack of storms also explains a substantial increase (∼30%) in solar radiation reaching the surface of western Patagonia during the summer of 2016 (Fig. 5a)^42^.

**Figure 6:**
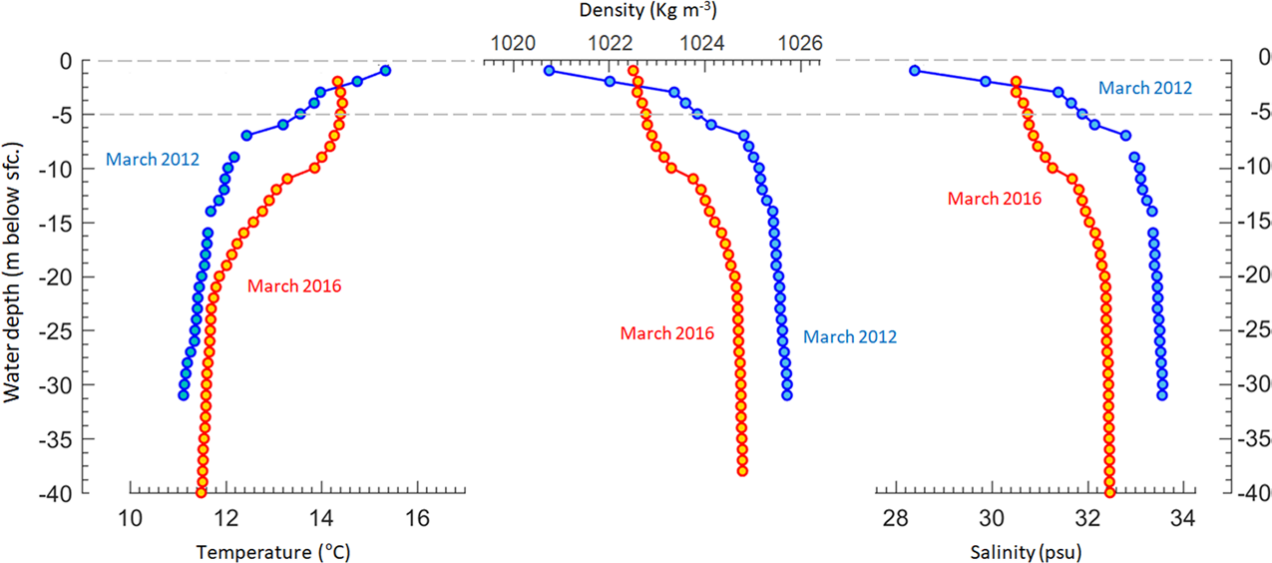
Water column stratification. Observed temperature (left panel) and salinity (right panel) in the Reloncaví Sound water column for 2012 (blue symbols, near normal streamflow) and March 2016 (red symbols filled in yellow, below normal streamflow). Also shown is the derived water density for both periods.

The low streamflow records during summer 2016 (Fig. 5b) in turn caused above normal surface salinity in Reloncaví Fjord and Sound, and hence weaker than normal haline stratification of the water column^18,49^. For comparison, Fig. 6 shows the temperature and salinity profiles in the Reloncaví Sound water column for March 2012 (when typical/normal streamflow was recorded, see Fig. 3b) and March 2016 (when very low streamflow was recorded). Under near normal streamflow (2012) there was a marked drop in salinity within the near surface layer (1–5 m) that was completely absent when the streamflow was low in 2016. Likewise, the thermocline was much sharper in 2012 compared to 2016. The lack of marked gradients in salinity and temperature during 2016 resulted in a smooth density increase downward, and hence a much weaker surface stratification in 2016 compared to a normal year (2012). Indeed, the mean Brunt Väisälä in the 1–5 m layer in 2016 was nearly half of that in 2012. Therefore, under the dry conditions of 2016 we expect deep (ocean) water to reach the surface rather frequently. When nutrient-rich waters reached the surface they received higher than normal solar radiation (Fig. 5a), generating optimum conditions for harmful phytoplankton species to bloom in the coastal waters of western Patagonia.

This weak stratification is also supported by direct observations from the coastal buoy in the Relconaví Fjord, revealing important changes in oceanographic parameters occurring during the summer/fall seasons (Fig. 4c). Surface warm waters (15–16 °C) with high dissolved oxygen (6–8 ml L^−1^) and relatively high salinity (25–27 psu) were observed prior to the bloom (February; Fig. 4c). By mid March there was an increase in salinity (up to 30 psu) and a gradual drop in temperature, probably linked to the lower river streamflow (Fig. 5b) and augmented frequency of saline and nutrient-rich deep water (Modified SubAntarctic Water, MSAAW)^56^ intrusions in the surface layer of the fjord.

The important role of reduced freshwater input and augmented insolation does not rule out other factors (e.g., upwelling-favorable winds) also contributing towards creating a favorable environment for HAB development. These and their relative contribution to the bloom need to be assessed on the basis of detailed hydrobiological modelling, sampling and study of other HAB events in this region.

### Conclusion and outlook

Although the worldwide occurrence of severe HABs in the last decades suggests a connection with anthropogenic climate change^7^, the causal link needs to be established at a regional scale^12^. Interaction between ocean and atmosphere at the global scale is complex, and heavily influenced by local dynamics as well. In the present study we demonstrate how several local and large scale factors and their interaction acted in concert to generate favourable conditions for the worst *Pseudochattonella* cf. *verruculosa* bloom ever recored. This bloom in inshore waters of western Patagonia during the 2016 austral summer caused major economic losses and sanitary risks in the Chilean Patagonia.

The strong El Niño 2015–2016 superimposed on the positive trend of SAM led to a marked reduction of the westerly flow impinging on the austral Andes and persistent anticyclonic conditions over the southeast Pacific and southern South America. These large-scale anomalies resulted in an extremely dry summer in western Patagonia, with record low streamflow and higher than normal solar radiation reaching the surface. The reduction in freshwater input was instrumental in the weakening of ocean stratification in the upper layer, thus allowing vertical advection of saline and nutrient-rich waters that ultimately resulted in the enhanced bloom of *Pseudochattonella* cf. *verruculosa*.

*Pseudochattonella* species have been reported to thrive in both relatively cold (2–5 °C) and in warm waters of the Northern Hemisphere (up to 18 °C)^25,27^. In southern waters of Patagonia, *Pseudochattonella* cf. *verruculosa* has achieved high cell abundance (4000–20000 cells mL^−1^) during higher temperatures (15–16 °C) and relatively high salinity (∼30 psu) (Figs 4c and 6), representative of oceanic water features during summer conditions. Furthermore, it has been pointed out that *Pseudochattonella* species formed HAB in the presence of high concentrations of silicic acid (>30 μM), even with enough nitrate and orthophosphate in the upper 20 m depth^25^. The marked reduction in freshwater input for the coastal zone for almost three months, together with the quantitative phytoplankton analyses and buoy data, show that the 2016 HAB developed under a weakly stratified water column dominated by relatively warm, salty and nutrient-rich deep water both in the inner sound and fjord areas. These oceanographic conditions coupled with the capacity of *Pseudochattonella* spp. to migrate vertically^26^ and the enhanced solar radiation reaching the surface may have favoured the growth and accumulation of *Pseudochattonella* cf. *verruculosa* in inner seas of Patagonia. The migration behavior of *P*. cf. *verruculosa* may have been related to nutrient uptake and selection of the optimal light environment at the pycnocline depth. The development of migration strategies by phytoplankton in variable environments subjected to pulsing dissolved nutrients could be advantageous given nutrient-deficient top surface layer conditions.

Although the 2016 HAB event seems more related to a large-scale climate-oceanographic forcing, we also acknowledge the potential influence of enhanced local nutrient input. Presently, the role of local nutrient pulses in stimulating blooms of specific algae as well as the spatial extent dynamics (offshore - onshore) of coastal blooms in northern Patagonia are not known. Indeed, the hypothesis connecting decrease in freshwater input and enhanced solar radiation triggering HABs emerges from the evidence gathered after the 2016 summer HAB in Patagonia and needs to be further validated with hydrobiological modeling and analysis of other events. Probing this hypothesis offers an opportunity to understand phytoplankton dynamics in Patagonia, and hence contribute to gain resilience towards strong HABs in the future.

The situation in Patagonia during the summer of 2016 (concomitant HAB development and dry conditions) bears a resemblance to the record-breaking 2015 diatom HAB along the west coast of North America^8^ as the inorganic nutrients in both systems are primarily supplied through vertical advection. Conversely, where the nutrient supply is primarily from land, an increase in rainfall/streamflow would result in increased nutrient loading^16^ and stratification^18^. This further highlights the need to understand the dynamics locally.

Given the high level of conservation of the river watersheds in remote areas in this part of Chile (little anthropogenic use), it would be useful to evaluate streamflow records accurately, as they might allow us to predict periods prone to the occurrence of anomalous bio-oceanographic events such as the 2016 HAB. Such evaluations would help to mitigate economic and ecosystem losses and could be a determining factor in the selection, planning, and development of future productive activities in coastal systems with strong freshwater influence. An integrative approach would help global aquaculture to gain resilience towards expected future changes. Understanding the association between climate anomalies, drought and HAB occurrence in western Patagonia is particularly relevant given the prospect of climate change in this region. Drier than present conditions are consistently projected for western Patagonia towards the end of the century^70^, as the increase in greenhouse gas will continue to shift the SAM toward its positive polarity, offsetting the recovery of stratospheric ozone^71^. Superposition of El Niño events in this altered climate may result in a higher frequency of extreme dry summers and perhaps environmental disruptions as observed in 2016.

### Data availability

The data sets generated during and/or analysed during the current study are available from the corresponding authors on reasonable request.
